# Higher precision and preservation of frontal plane alignment in slope‐reducing infra‐tubercle compared to retro‐tubercle high tibial osteotomy

**DOI:** 10.1002/ksa.70028

**Published:** 2025-09-05

**Authors:** Romir Patel, Ahmed Mabrouk, Kristian Kley, Christophe Jacquet, Lucas Abdelkafi, Théo Plassard, Matthieu Ollivier

**Affiliations:** ^1^ Department of Orthopedic Surgery, APHM, CNRS, ISM, Institute of Movement Sciences, Sainte‐Marguerite Hospital Aix Marseille University Marseille France; ^2^ Trauma and Orthopaedics Department Basingstoke and North Hampshire Hospitals Basingstoke UK; ^3^ Orthopaedic Care Center Harley Street Specialist Hospital London UK

**Keywords:** ACL injuries, frontal alignment, multi‐ligament knees, osteotomy, posterior tibial slope

## Abstract

**Purpose:**

Slope‐reducing high tibial osteotomies (SR‐HTOs) correct posterior tibial slope (PTS) abnormalities in patients with anterior knee instability, as in cases of anterior cruciate ligament (ACL) deficiency. The SR‐HTO techniques, including infra‐tubercle and retro‐tubercle approaches, provide distinct benefits: retro‐tubercle techniques help preserve patellofemoral joint mechanics, while infra‐tubercle techniques are effective in mitigating iatrogenic varus. However, there is limited comparative literature available. This study compares the PTS correction precision, frontal plane alignment changes, patellar height (PH) alterations and complications between both SR‐HTO techniques.

**Methods:**

A retrospective matched cohort study including 62 patients who underwent SR‐HTO with ACL revision surgery between 2020 and 2023 was conducted. Of the 62 patients included, 40 (64.5%) were male and 22 (35.5%) were female. The mean follow‐up period was 23.4 ± 7.7 months (range: 12–45 months). The cohort was subdivided into infra‐tubercle (*n* = 29) and retro‐tubercle (*n* = 33) groups. Preoperative and post‐operative radiographic assessments included hip–knee–ankle angle (HKA), medial proximal tibial angle (MPTA), proximal tibial slope (PTS) and PH indices. Functional outcomes were measured using the simple knee value (SKV) score. Complications such as hinge fractures, ACL re‐rupture and hardware removal were recorded.

**Results:**

Both techniques achieved similar mean slope correction with post‐operative PTS in infra‐tubercle: 9.2 ± 1.1° (range: 5–13.8°) versus retro‐tubercle: 9.1 ± 1.3° (range: 5–14°). This has been reduced from a preoperative PTS in the infra‐tubercle group of 14.2 ± 1.7° (range: 11.5–17°) versus retro‐tubercle group: 14 ± 1.8° (range: 11–17.5°). Infra‐tubercle osteotomy showed greater precision to pre‐operative plans, with a deviation of 1.2 ± 1.1° (range: 0.0–3.6°) versus 1.8 ± 1.3° (range: 0.0–3.4°) in the retro‐tubercle group (*p* = 0.02). Retro‐tubercle SR‐HTO induced greater coronal changes compared to infra‐tubercle SR‐HTO, ΔHKA: 1.4 ± 1.6° (range: 0–5°) versus 0.8 ± 0.8° (range: 0–2.8°) (*p* = 0.05); ΔMPTA: 1.6 ± 1.6° (range: 0–5.7°) versus 0.9 ± 0.7° (range: 0–2.8°) (*p* = 0.03). There was no intergroup difference in PH changes using either Caton–Deschamps or Schroter indices (*p* = 0.2). SKV improvement was greater in the infra‐tubercle group compared to the retro‐tubercle group, 28.7 ± 10.4 (range: 10.0–55.7) versus 20.7 ± 12.3 (range: −9.2 to 48.4) (*p* = 0.008). Complications were similar, with no hinge fractures and identical ACL re‐rupture rates of 3.4%. Hardware removal was higher in the infra‐tubercle group compared to the retro‐tubercle group, 24.1% versus 9.1% (*p* = 0.2).

**Conclusion:**

Infra‐tubercle SR‐HTO demonstrated greater correction accuracy and better preservation of frontal plane alignment and functional outcomes compared to the retro‐tubercle technique, although the observed differences were modest. Both techniques maintained PH and exhibited comparable safety profiles. Infra‐tubercle SR‐HTO may offer a reliable alternative, particularly in ACL‐deficient knees where precise slope correction is desired.

**Level of Evidence:**

Level III, retrospective comparative study.

**Abbreviations**
ACL: anterior cruciate ligament
ACLR: anterior cruciate ligament reconstruction
ACW: anterior closing‐wedge
ACW‐HTO: anterior closing‐wedge high tibial osteotomy
ANOVA: analysis of variance
ATT: anterior tibial translation
CDI: Caton–Deschamps index
EHR: electronic health record
GE: General Electric
HKA: hip–knee–ankle angle
HTOs: high tibial osteotomies
IBM: international business machines
ICC: intraclass correlation coefficient
MPTA: medial proximal tibial angle
PACS: picture archiving and communication system
PH: patellar height
PPTA: proximal posterior tibial angle
PTS: posterior tibial slope
ROM: range of motion
SD: standard deviation
SKV: simple knee value
SPSS: statistical package for social sciences
SR‐HTO: slope‐reducing high tibial osteotomy
ΔHKA: mean change in hip–knee–ankle angle
ΔPTS: mean change in posterior tibial slope

## INTRODUCTION

Slope‐reducing high tibial osteotomies (SR‐HTOs) are a key surgical procedure for correcting the posterior tibial slope (PTS) in patients with knee instability, especially those with anterior cruciate ligament (ACL) deficiency [1, 20, 26]. Altering the PTS helps restore knee stability and prevents excessive tibial translation and consequent ACL reconstruction failure [26].

SR‐HTO are performed using either the retro‐tubercle or infra‐tubercle technique. The retro‐tubercle technique, in which the osteotomy is made posterior to the tibial tubercle, preserves patellofemoral joint mechanics and patellar height (PH) [6, 12]. However, it presents challenges in slope control due to limited visualization and an unfavourable osteotomy angle, with higher risks of non‐union, tuberosity fractures, and tibial tuberosity prominence [6, 12]. Additionally, iatrogenic varus is a common post‐operative issue [3]. In contrast, the infra‐tubercle approach, performed distal to the tibial tuberosity, allows greater PTS correction while maintaining patellar tendon insertion [24]. The infra‐tubercle technique preserves extensor mechanism integrity and patellofemoral kinematics, preventing patella alta or baja [7, 11, 27]. Moreover, it mitigates iatrogenic varus, making it particularly advantageous for ACL‐deficient knees compared to the retro‐tubercle approach [17, 24, 25, 26].

Despite the benefits of both osteotomies, there is limited comparative research in the current literature. The infra‐tubercle approach is a relatively new technique and has shown promising results in small cohorts [24, 25], but larger comparative studies are needed to confirm its advantages over more traditional osteotomies to correct PTS and limit complications.

This study presents the first comparative analysis between the infra‐tubercle and retro‐tubercle SR‐HTO techniques. The primary objectives are to compare the degree of angular correction, the precision of PTS correction, changes in frontal plane alignment (using hip–knee–ankle angle [HKA] and medial proximal tibial angle [MPTA]), and PH alterations post‐operatively. Secondary outcomes include functional outcomes as measured by simple knee value (SKV), and the incidence of post‐operative complications, including hinge fractures, ACL re‐rupture, and hardware removal rates. It was hypothesized that the infra‐tubercle SR‐HTO technique would outperform the retro‐tubercle technique in PTS correction precision, angular correction and frontal plane alignment, with lower complications and better functional outcomes.

## METHODS

### Study design and patient selection

After institutional review board approval, a retrospective comparative study was conducted at a single institution, including patients who underwent revision ACL reconstruction (ACLR) with concurrent slope‐reducing osteotomy between January 2020 and January 2023. All surgical procedures were performed by a single surgeon (M.O.). A total of 97 consecutive adult patients were identified through electronic health records (EHRs). The inclusion criteria comprised patients undergoing slope‐reducing osteotomy for ACL‐deficient knees, with either single‐ or double‐stage ACLR depending on tunnel width, the availability of long‐axis radiographs, and a complete dataset within the EHR. In cases undergoing two‐stage ACL revision, the average time interval between stages was 6 ± 3 months. The decision to proceed with one‐ versus two‐stage procedures was based on preoperative tunnel evaluation: a single‐stage procedure was performed when the tunnel was in an acceptable position, the tunnel showed no significant ballooning, and did not require grafting. The exclusion criteria encompassed patients with multi‐ligamentous knee injuries, posterior tibial fractures, slope‐increasing osteotomies and those requiring frontal plane corrections.

### Surgical technique

**Figure 1:**
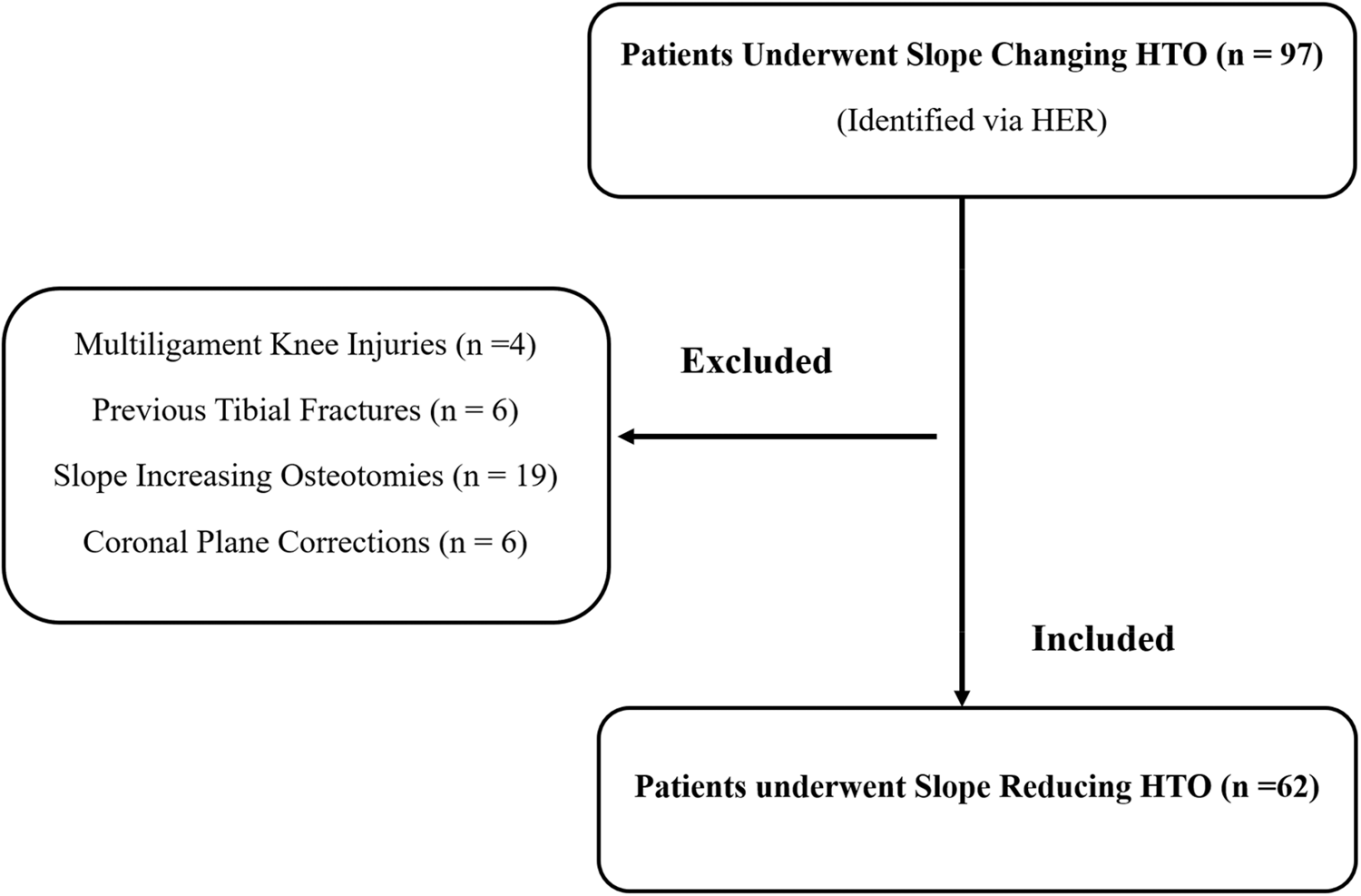
Flowchart describing patient selection. HTO, high tibial osteotomy.

The infra‐tubercle anterior closing‐wedge high tibial osteotomy (ACW‐HTO) (Figure 1) was performed as described previously [9, 24], where a medial longitudinal incision was made 3 cm distal to the tibial tuberosity, with subperiosteal dissection preserving the patellar tendon. K‐wires guided the anterior and posterior osteotomy cuts, which were made distal to the tibial tubercle to protect the extensor mechanism. After removing the anterior wedge, axial compression was applied to close the site in full extension, achieved by using a 6‐hole locking plate.

**Figure 2:**
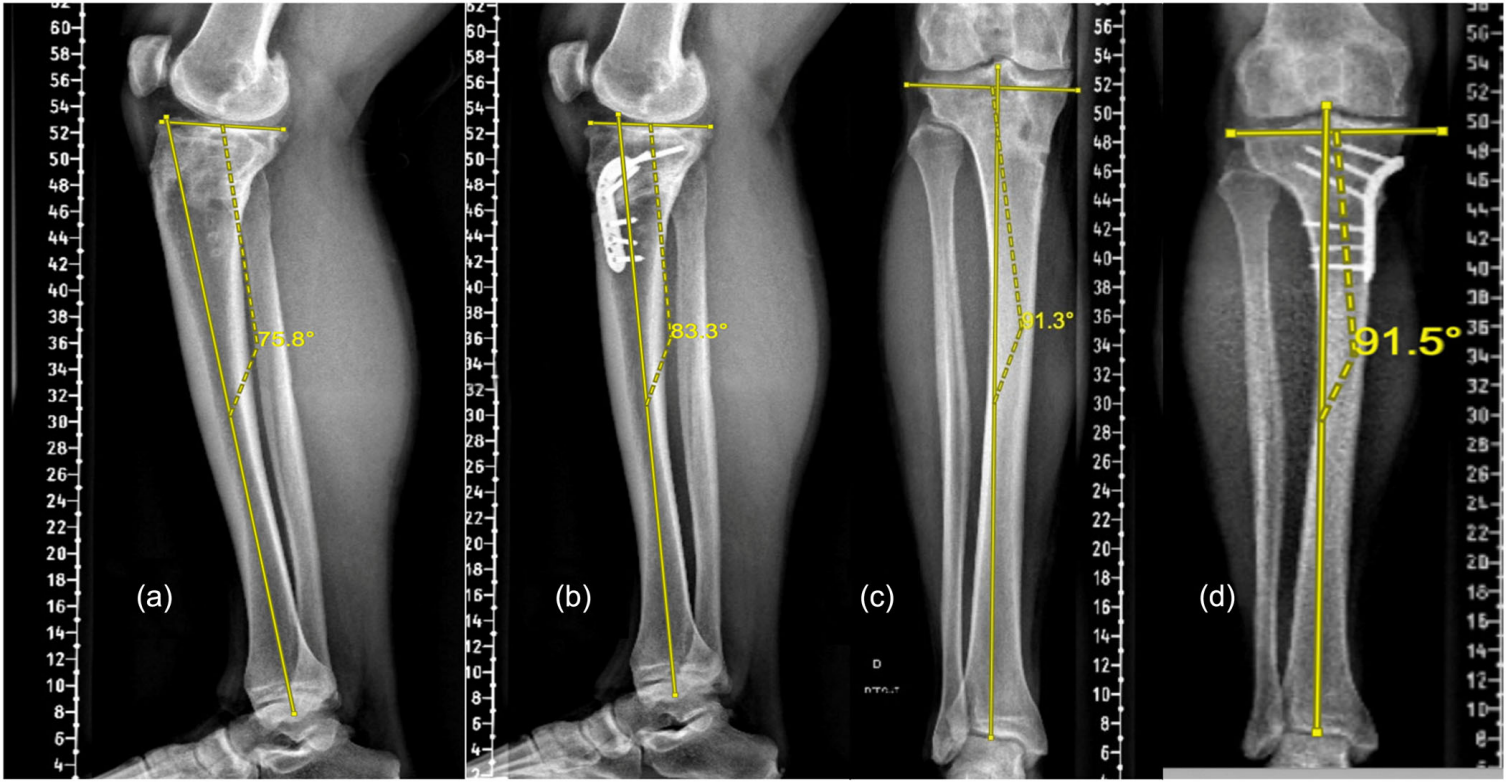
Preoperative and post‐operative x‐rays showing an infra‐tubercle anterior closing wedge osteotomy compressed using a locking plate (Active‐motion S plate, Newclip technique). (a) Preoperative lateral view. (b) Post‐operative lateral view. (c) Preoperative frontal view. (d) Post‐operative frontal view.

The retro‐tubercle closing‐wedge osteotomy (Figure 2) was performed following a previously described technique [8]. A midline incision was made to expose the tibial tubercle. K‐wire‐guided cuts were made behind the tibial tubercle, followed by four ascending cuts towards the posterior cortex with protective K wires to prevent hinge fractures. Then, the bone wedge was removed, axial compression was applied to close the site in full extension, achieved by using 6‐hole locking plate.

The choice between infra‐ and retro‐tubercle osteotomy was based on the surgeon's preference. Retro‐tubercle osteotomy was typically selected in cases requiring more extensive slope correction due to its capacity for greater resection height. Furthermore, intraoperative alignment in both the sagittal and coronal planes was verified using fluoroscopy to ensure proper correction and prevent unintended malalignment.

### Post‐operative protocol

All patients in both groups followed a similar post‐operative rehabilitation. Portable post‐operative radiographs were taken in recovery to confirm the alignment and hardware position. Early physiotherapy management was focused on pain control and swelling reduction. All patients had a knee immobilizer with the knee in full extension in the first week, followed by a progressive increase in the ROM from 0° to 90° between Weeks 1 and 3 post‐operatively. Patients were instructed to remain non‐weight‐bearing for 3 weeks with the help of crutches. After the third week, patients were allowed full weight‐bearing and full ROM as tolerated. Solid osteotomy union and bone tunnel healing were recommended in cases where a two‐stage ACL revision was planned. Patients were serially followed up at 6 weeks, 3 months, 6 months, 12 months and yearly thereafter.

### Radiographic assessment

**Figure 3:**
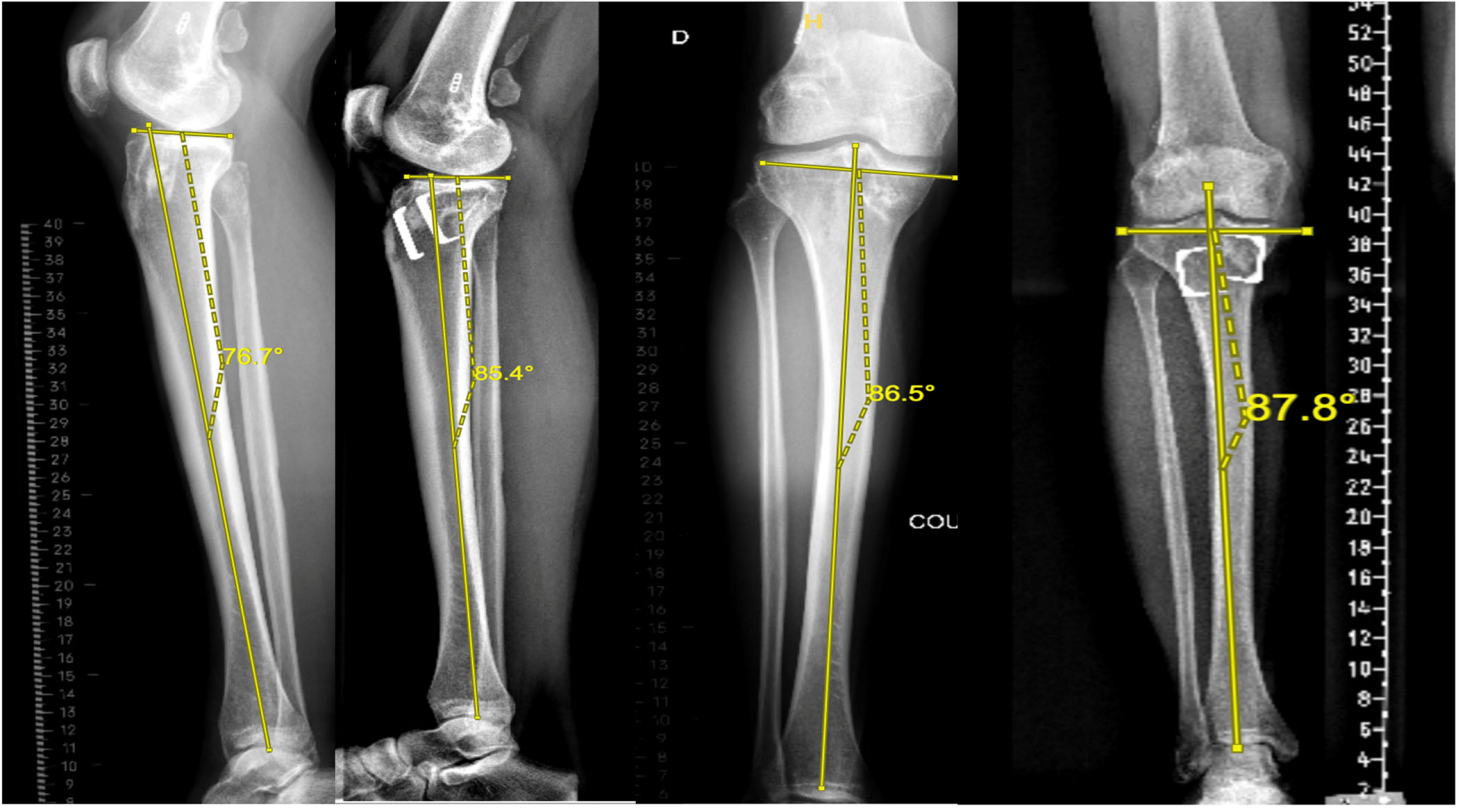
Preoperative and post‐operative full‐length tibia radiographs demonstrating a retro‐tubercle anterior closing wedge osteotomy stabilised using two clips. (a) Preoperative lateral view. (b) Post‐operative lateral view. (c) Preoperative frontal view. (d) Post‐operative frontal view.

**Figure 4:**
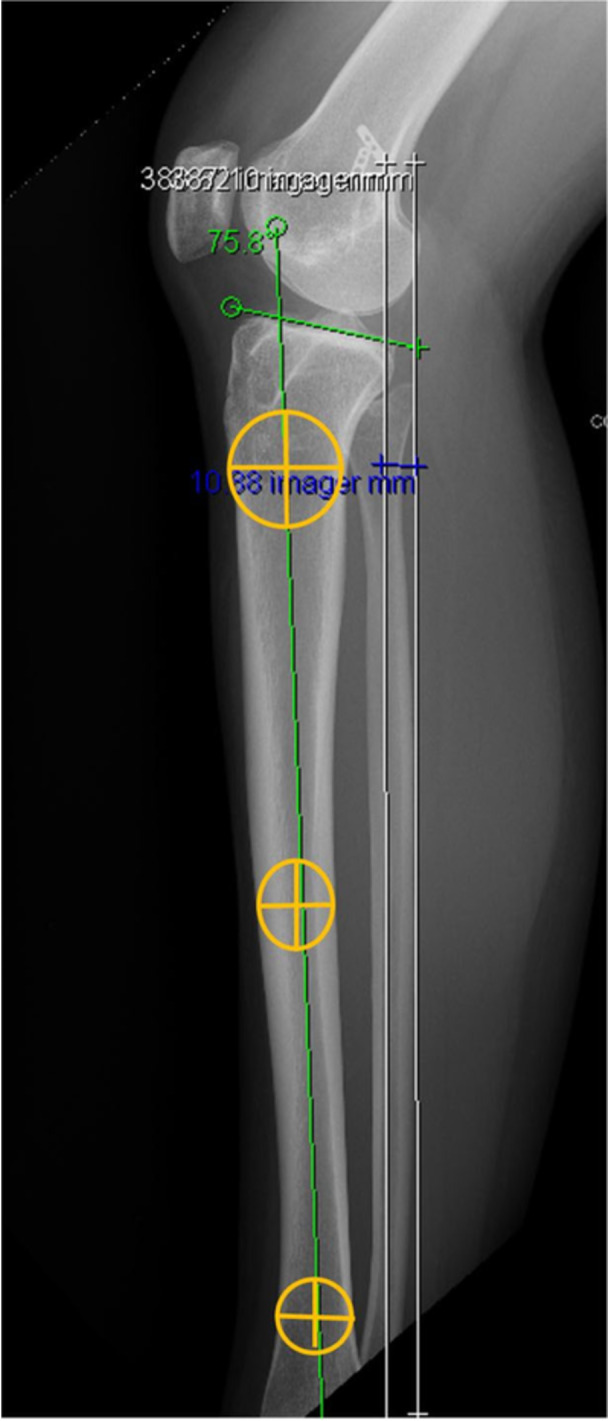
Three‐circle method to determine posterior tibial slope.

Full‐length lower limb x‐rays were used to assess the HKA and MPTA (Figure 3). The proximal posterior tibial angle (PPTA) was used as a representative of the PTS, which was measured on a lateral knee radiograph as the angle between the posterior inclination of the medial tibial plateau and the tibial longitudinal axis. The longitudinal axis of the tibia was determined using the circle three‐point method as outlined in previous studies [13, 14, 19, 23]. This included drawing two circles at 90 and 150mm distal to the joint line, centred within the tibial diaphysis and a third circle at the distal tibial diaphysis. The perimeters of the circles were aligned with the anterior and posterior borders of the tibial shaft. The line connecting the centres of these two circles was then used to define the longitudinal axis of the tibia (Figure 4).

**Figure 5:**
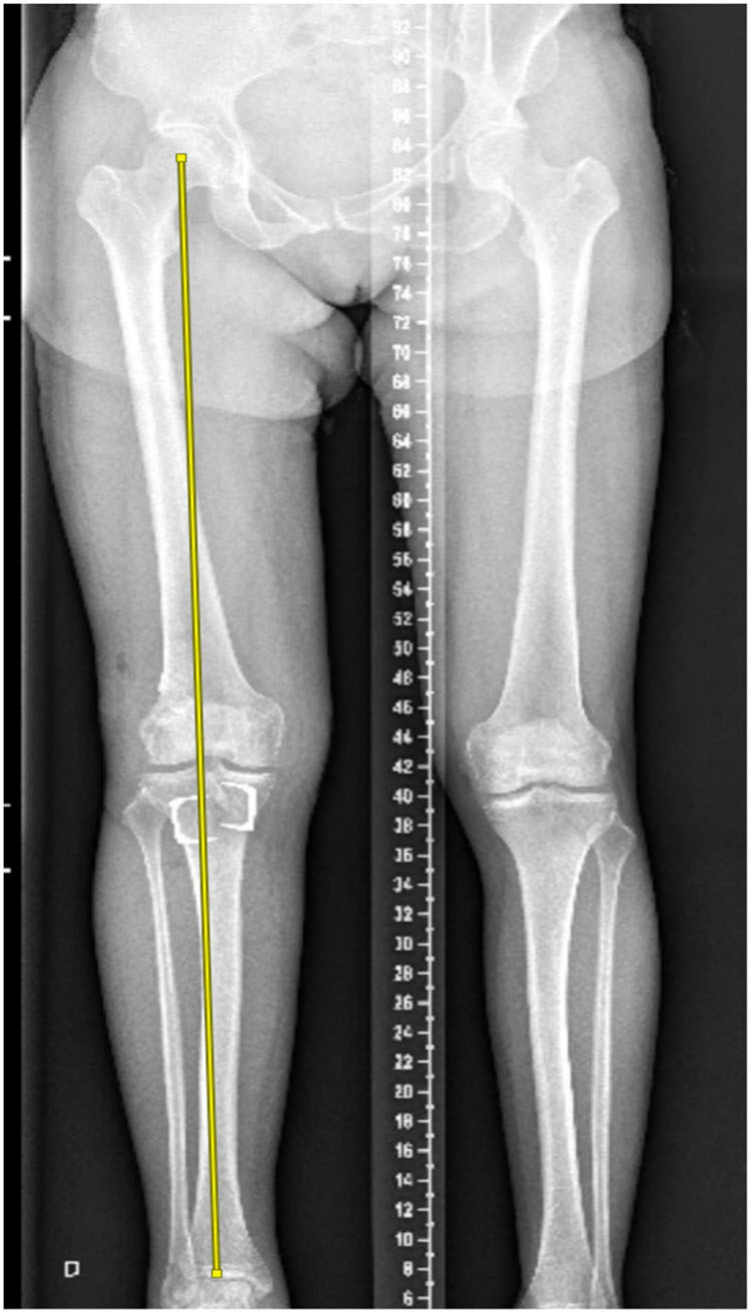
Long leg standing radiographs demonstrating Mikucliz line indicating neutral coronal alignment of the limb.

To ensure accurate measurement and minimize variability due to malrotation, only lateral radiographs with posterior condyle overlap <5 mm and distal condyle overlap <5 mm were included, in line with established radiographic quality standards (Figure 5).

Preoperative PH was evaluated on lateral radiographs using two measurements relative to the proximal tibia: the Caton–Deschamps index (CDI) [4] and the femur‐referenced index, the Schröter index [10] was assessed on anteroposterior knee radiographs.

### Post‐operative clinical and radiographic assessment

Patient characteristics, operative side, number of ACL revisions, age, body mass index and sex were extracted from EHR. The following pre‐operative and post‐operative alignment indices were identically recorded, including the HKA, MPTA, PTS, and PH. The mean difference (Δ) PTS was defined as the difference between post‐operative PTS and pre‐operative PTS. Delta objective PTS (°) was defined as the difference between planned PTS and actual PTS as measured on radiographs. Clinically, pre‐ and post‐operative SKV [21] scores were extracted from EHR. Post‐operative complications—Hinge fractures, ACL rupture and hardware removal—were extracted from EHR. Furthermore, bony union was assessed using standard radiographs obtained monthly for the first 6 months post‐operatively, and every 6 months thereafter.

### Statistical analysis

All statistical analyses were conducted using SPSS (version 20.4, IBM Corp.). The mean difference (Δ) between preoperative and follow‐up parameters was calculated. Variables included lower limb alignment parameters (HKA, MPTA and PTS), PH measurement parameters (CDI and Schröter index) and functional outcomes (SKV). Continuous variables, including HKA, PTS and SKV, were analyzed using ANOVA and paired *t* tests to assess differences between preoperative and post‐operative values. Categorical variables, such as PH classifications and complication rates, were analyzed using chi‐squared tests and Fisher's exact tests. Results were reported with 95% confidence intervals, and statistical significance was defined as *p* < 0.05.

Radiographic measurements were conducted using the Centricity PACS software (GE Healthcare). All assessments were performed by two independent investigators (M.O. and A.M.). To evaluate measurement reliability, radiographs were reanalyzed by the same investigator three weeks after the initial assessment. The intraclass correlation coefficient (ICC) was 0.91 for intra‐observer reliability and 0.82 for inter‐observer reliability, measured overall for all radiographic measurements (Tables 1 and 2). A post hoc power analysis was performed to confirm the adequacy of the sample size. To detect a clinically meaningful difference of >1° in radiographic angular measurements (HKA, MPTA and PTS), assuming an expected standard deviation of 1.5°, a minimum of 29 patients per group was required to achieve 80% statistical power (*α* = 0.05). This analysis confirmed the study was adequately powered to detect both within‐group (pre‐ vs. post‐operative) and between‐group (infra‐ vs. retro‐tubercle) differences.

**Table 1:** Intraobserver intraclass correlation coefficient (ICC) for all radiographic measurements.

Statistic	Value
ICC (absolute agreement)	0.89
Confidence interval (95%)	0.81–0.94
*p*	<0.001
Within‐subject variance	0.3
Number of subjects	62

**Table 2:** Interobserver intraclass correlation coefficient (ICC) for all radiographic measurements.

Statistic	Value
ICC (absolute agreement)	0.80
Confidence interval (95%)	0.69–0.91
*p*	<0.001
Within‐subject variance	0.4
Number of subjects	62

## RESULTS

A total of 62 patients were included in the study, with 29 in the infra‐tubercle group and 33 in the retro‐tubercle group. Baseline demographics and preoperative alignment measures were comparable between groups (Table 3).

**Table 3:** Demographics and preoperative alignment.

Parameter	Retro‐tubercle	Infra‐tubercle	*p* **value**
Age (years)	20.4 ± 3.5 (range: 16–31)	21.8 ± 3.1 (range: 16–31)	0.2
BMI (kg/m^2^)	20.9 ± 2.5 (range: 19–28)	22.8 ± 2.4 (range: 19–28)	0.3
Sex (% female)	36.4%	34.5%	0.9
Follow‐up (months)	26.7 ± 8.2 (range: 12–45)	19.6 ± 5.0 (range: 12–33)	0.0001
Two‐stage procedure (*n*)	2	3	
HKA preoperative (°)	178.8 ± 2.1 (range: 175–185)	178.1 ± 1.7 (range: 175–181)	0.1
MPTA preoperative (°)	85.0 ± 1.8 (range: 82–89)	84.2 ± 1.1 (range: 82–86)	0.07
PTS preoperative (°)	14.0 ± 1.8 (range: 11–18)	14.2 ± 1.7 (range: 11–18)	0.7
ACL revisions (total)			0.40
1 revision	0 (0%)	1 (3.4%)	
2 revisions	12 (36.4%)	12 (41.4%)	
3 revisions	20 (60.6%)	16 (55.2%)	
4 revisions	1 (3.0%)	0 (0%)	

Abbreviations: ACL, anterior cruciate ligament; BMI, body mass index; HKA, hip–knee–ankle angle; MPTA, medial proximal tibial angle; PTS, proximal tibial slope.

The mean change in PTS (ΔPTS) was similar between the infra‐tubercle (9.2 ± 1°, target 10.4 ± 2.7°) and retro‐tubercle (9.1 ± 1°, target 10.9 ± 2.5°) groups (n.s.). However, infra‐tubercle osteotomy demonstrated significantly greater precision relative to the preoperative plan, with a deviation of 1.2 ± 1° compared to 1.8 ± 1° in the retro‐tubercle group (*p* = 0.02) (Table 4). Preoperative planning was conducted using PEEKMED software with the three‐circle method, referencing the medial tibial slope. The typical target PTS was 5–6°, with a preference for 6° in cases requiring greater correction.

**Table 4:** PTS change and objective delta PTS.

Parameter	Retro‐tubercle	Infra‐tubercle	*p* **value**
Actual PTS change (°)	9.1 ± 1.3 (range: 6.0–12.0)	9.2 ± 1.1 (range: 6.8–11.6)	NA
Delta objective PTS (°)	1.8 ± 1.3 (range: 0.0–3.6)	1.2 ± 1.1 (range: 0.0–3.4)	0.02
Planned PTS correction (°)	10.9 ± 2.4 (range: 5.8–16.6)	10.4 ± 2.7 (range: 5.3–16.3)	NA
Post‐operatitve PTS (°)	85.2 ± 1.2 (range: 82–89)	85.1 ± 1.4 (range: 83–89°)	

Abbreviation: PTS, proximal tibial slope.

The retro‐tubercle group exhibited significantly greater coronal plane changes. The ΔHKA was 1.4 ± 1.6° in the retro‐tubercle group compared to 0.8 ± 0.8° in the infra‐tubercle group (*p* = 0.0466). To further characterize these changes, ΔHKA was categorized as varus shift (ΔHKA < −0.5°), valgus shift (ΔHKA > +0.5°) or neutral (−0.5° to +0.5°). In the retro‐tubercle group, 60.6% (*n* = 20) of patients experienced a varus shift, 3.0% (*n* = 1) a valgus shift and 36.4% (*n* = 12) remained neutral. In the infra‐tubercle group, 34.5% (*n* = 10) shifted towards varus, 20.7% (*n* = 6) towards valgus and 44.8% (*n* = 13) remained within the neutral range. This change in alignment differed significantly between groups (*p* = 0.0348, chi‐square test).

Similarly, the mean change in MPTA (ΔMPTA) was 1.6 ± 1.5° in the retro‐tubercle group versus 0.9 ± 0.7° in the infra‐tubercle group (*p* = 0.03) (Table 5).

**Table 5:** Post‐operative alignment and changes.

Parameter	Retro‐tubercle	Infra‐tubercle	*p* **value**
HKA post‐operative (°)	177.5 ± 1.8 (range: 173–181)	177.9 ± 2.3 (range: 173–181)	0.5
ΔHKA (°)	1.4 ± 1.6 (range: 0–5)	0.8 ± 0.8 (range: 0–4)	0.0466
Coronal alignment change			0.0348
Varus	60.6% (*n* = 20)	34.5% (*n* = 10)	
Neutral	36.4% (*n* = 12)	44.8% (*n* = 13)	
Valgus	3.0% (*n* = 1)	20.7% (*n* = 6)	
MPTA post‐operative (°)	83.7 ± 1.8 (range: 81–88)	83.9 ± 1.2 (range: 81–86)	0.7
ΔMPTA (°)	1.6 ± 1.5 (range: 0–8)	0.9 ± 0.7 (range: 0–5.7)	0.0347

Abbreviations: HKA, hip–knee–ankle angle; MPTA, medial proximal tibial angle.

There was no significant difference in PH changes between groups. PH alteration occurred in seven patients (21.2%) in the retro‐tubercle group and two patients (6.9%) in the infra‐tubercle group (*p* = 0.2), while not statistically significant, this may have clinical relevance (Table 6).

**Table 6:** Patellar height metrics.

Parameter	Retro‐tubercle	Infra‐tubercle	*p* **value**
Patellar height change			0.1
No change	77.8%	93.1%	
Increased	22.2%	6.9%	
CD Index—Preoperative	1.1 ± 0.2 (range: 0.8–1.5)	1.2 ± 0.1 (range: 1.0–1.5)	0.009
CD Index—Post‐operative	1.3 ± 0.1 (range: 1.0–1.5)	1.2 ± 0.1 (range: 1.1–1.4)	<0.0001
ΔCD (Post–Pre)	0.2 ± 0.2 (range: −0.2 to 0.3)	−0.01 ± 0.1 (range: −0.2 to 0.3)	<0.0001
Schroeter Index—Preoperative	1.15 ± 0.13 (range: 0.9–1.3)	1.17 ± 0.11 (range: 1.0–1.3)	0.5
Schroeter Index—Post‐operative	1.15 ± 0.13 (range: 1.0–1.4)	1.25 ± 0.09 (range: 1.1–1.4)	0.0014
ΔSchroeter (Post–Pre)	0.01 ± 0.09 (range: −0.2 to 0.3)	0.09 ± 0.12 (range: −0.2 to 0.3)	0.0054

Abbreviation: CD, Caton–Deschamps.

Complication rates were similar in both groups. The infra‐tubercle group had a higher rate of hardware removal and an earlier time to removal, seven patients at 9 months versus three patients at 12.3 months, though not statistically significant (*p* = 0.1672 and *p* = 0.1355, respectively). Furthermore, Re‐rupture rates were identical at one patient in both groups, with a time to re‐rupture of 13 months in the infra‐tubercle group and 14 months in the retro‐tubercle group (Table 7). There were no hinge fractures in either group (Table 8).

**Table 7:** Pre‐ and post‐operative simple knee value (SKV) scores in both groups.

Parameter	Retro‐tubercle	Infra‐tubercle	*p* **value**
SKV preoperative (°)	44.1 ± 10.0 (range: 20.0–68.8)	48.6 ± 13.0 (range: 20.0–68.8)	0.1
SKV post‐operative (°)	64.8 ± 9.4 (range: 38.8–91.0)	77.3 ± 11.7 (range: 38.8–91.0)	<0.0001
ΔSKV (°)	20.7 ± 12.3 (range: −9.2 to 55.7)	28.7 ± 10.4 (range: −5.8 to 55.7)	0.008

**Table 8:** Complications: Hardware removal, ACL re‐rupture and times.

Parameter	Retro‐tubercle	Infra‐tubercle	*p* **value**
Hardware removal rate (*n* (%))	3 (9.1)	7 (24.1)	0.17
Time to hardware removal (months)	12.3 ± 1.6 (range: 11–13)	9.0 ± 1.1 (range: 6–14)	0.14
ACL re‐rupture rate (*n* (%))	1 (3.0)	1 (3.4)	1
Time to ACL re‐rupture (months)	13.0	14.0	1

Abbreviation: ACL, anterior cruciate ligament.

## DISCUSSION

The key finding of the present study is that infra‐tubercle SR‐HTO demonstrates statistically significantly greater precision in PTS reduction and functional outcomes while preserving frontal plane alignment compared to retro‐tubercle SR‐HTO. Additionally, both techniques were equally effective in maintaining PH and had low rates of complications.

PTS is a critical determinant of ACLR outcomes, with increased PTS predisposing patients to greater anterior tibial translation (ATT) and higher rates of ACL graft failure [2, 15, 16, 31]. While both osteotomy techniques investigated in this study effectively reduced PTS, the infra‐tubercle approach demonstrated significantly greater precision in achieving the targeted slope reduction as determined during preoperative planning. These findings are consistent with existing literature supporting the efficacy of infra‐tubercle osteotomies in PTS reduction. Onishi et al. reported a mean PTS reduction of 8.8° using this technique [25]. Similarly, the present study observed a slightly greater mean reduction of 9.2°, further reinforcing the reliability and consistency of this approach.

Maintaining frontal plane alignment is crucial during osteotomy procedures to prevent complications associated with malalignment. Many previous studies have exhibited that ACWO can lead to decreased MPTA in the frontal plane, causing unintentional varus [22, 30]. This can lead to further complications in patients with multiple ACLR failures as they are more susceptible to medial chondral injuries [5], thus important to avoid varus alignment. In this study, the infra‐tubercle technique preserved frontal plane alignment more effectively than the retro‐tubercle technique, which induced significantly larger changes in HKA and MPTA. Onishi et al. [25] recently reported a mean increase in the HKA of 0.9° in their cohort, closely matching the 0.76° increase observed in our study. Similarly, Cance et al. [3] demonstrated that retro‐tubercle slope‐reducing osteotomies in ACLR patients significantly increased MPTA measurements by 0.95° towards varus alignment, which is consistent with our findings. Though statistically significant, it remains to be studied if this <1° iatrogenic varus induction is clinically significant, as experts have suggested >5° of coronal malalignment can affect ACLR outcomes [28].

PH changes are a major concern in knee osteotomies and have been extensively studied. In this study, both techniques effectively preserved PH, with no significant differences observed between the groups. This finding aligns with existing literature on osteotomies. Onishi et al. [25] reported that 14.3% of patients in their infratubercle ACWO group had a higher patella, compared to 6.9% in our study based on CDI values. Similarly, Cance et al. [3] observed a significant increase in PH, consistent with our results. A possible explanation for this CDI‐measured increase is the reduction in ATT following PTS correction [18, 26, 29]. Decreasing the PTS reduces ATT, increasing the patellar‐tibial distance. Since CDI is calculated as the distance between the patella's inferior pole and the anterior tibial plateau, this may artificially inflate CDI values, highlighting its potential limitations in this context.

Several limitations exist in the presented study, including the retrospective nature of the study and the relatively small sample size, which limit the generalizability of these findings. Furthermore, the shorter follow‐up time in the infra‐tubercle group may underestimate late complications or alignment changes. Radiological measurements—PTS, HKA and MPTA—were evaluated, and variability in interpretations between readers could influence results. Despite these limitations, this study represents the first of its kind to directly compare retro‐tubercle and infra‐tubercle osteotomies in the context of ACL reconstruction, PTS correction, and their relationship to both radiological and functional outcomes.

## CONCLUSION

Infra‐tubercle SR‐HTO demonstrated greater correction accuracy and better preservation of frontal plane alignment and functional outcomes compared to the retro‐tubercle technique, although the observed differences were modest. Both techniques maintained PH and exhibited comparable safety profiles. Infra‐tubercle SR‐HTO may offer a reliable alternative, particularly in ACL‐deficient knees where precise slope correction is desired.

## AUTHOR CONTRIBUTIONS

All authors contributed equally to the conception, design, data collection, analysis, drafting and critical revision of this manuscript. All authors approved the final submitted version.

## CONFLICT OF INTEREST STATEMENT

Kristian Kley and Matthieu Ollivier are paid consultants and receive royalties from Newclip. Matthieu Ollivier is also a paid consultant and receives royalties from Stryker. The remaining authors declare no conflicts of interest.

## ETHICS STATEMENT

Ethics approval was obtained from the Institute of Movement and Musculoskeletal System, Department of Orthopaedic Surgery of Sainte‐Marguerite Hospital, France (Reference: 2015‐A00133; Committee reference: 05,10 Report Number: 2015‐10‐00148). Informed consent was obtained from all patients involved in this study.

## Data Availability

All data used in this study are available from the corresponding author upon reasonable request.
